# Fever or hypothermia following ECMO decannulation: the association of body temperature with survival

**DOI:** 10.1186/s13054-023-04790-2

**Published:** 2024-01-04

**Authors:** Markus Busch, Benjamin Seeliger, Jan Fuge, Marius M. Hoeper, Klaus Stahl, for the Post-ECMO-Fever Research Group, Christian Kühn, Julius Schmidt, Nina Rittgerodt, Christine Fegbeutel, Olaf Wiesner, Heiner Wedemeyer

**Affiliations:** 1 Department of Gastroenterology, Hepatology, Infectious Diseases and Endocrinology, Hannover Medical School, Carl-Neuberg-Str. 1, 30625 Hannover, Germany; 2 Department of Respiratory Medicine, Hannover Medical School, Hannover, Germany; 3 Biomedical Research in End-Stage and Obstructive Lung Disease (BREATH), Hannover Medical School (MHH), German Center for Lung Research (DZL), Hannover, Germany

**To the Editor,**

Extracorporeal membrane oxygenation (ECMO) provides temporary circulatory or pulmonary support in the setting of refractory cardiogenic shock or respiratory failure, but weaning from ECMO remains challenging and carries a remarkable risk of failure [1, 2]. Decannulation from ECMO is often followed by fever [3] and less frequently by hypothermia, but it is unknown whether fever or hypothermia after decannulation influence survival or length of stay.

In this retrospective large single center observational study, body temperature during ECMO-weaning was analyzed in 223 consecutive patients from January 2016 to January 2019. 139 (62%) patients were treated on a cardiosurgical intensive care unit and 84 (38%) on a medical intensive care unit. 71 patients (32%) were supported by vv-ECMO, 149 (67%) by va-ECMO and 3 (1%) by veno-venous-arterial (vva)-ECMO. Patients were followed to discharge from Intensive Care Unit (ICU) or death. Fever and hypothermia were defined as core body temperature of above 38 °C and below 35 °C, respectively, occurring within 72 h following ECMO decannulation. Influence of post-ECMO-fever and hypothermia on ICU mortality was analyzed by competing risk regression models. Potential risk factors for post-ECMO-fever and hypothermia were identified by conducting multivariate logistic regression analyses.

139 of 223 patients (62%) developed fever within the first 72 h following ECMO explantation. In 107 patients (80%), fever occurred within the first 24 h following explantation, the median (interquartile range (IQR)) fever duration was 71 h (22–168) and the median spike temperature was 38.8 °C (IQR 38.3–39.3). In contrast, only 23 of 223 patients (10%) developed hypothermia within the first 72 h after explantation.

ICU mortality was not different between patients with (15.8%, n = 22/139) and without (11.9%, n = 10/84) fever following ECMO decannulation (Fig. 1A**/**B). In contrast, ICU mortality was 39.1% in patients with hypothermia (n = 9/23), while it was only 11.5% (n = 23/200) in patients not experiencing hypothermia (Fig. 1C**/**D). While post-ECMO-fever was not associated with increased ICU mortality (adjusted subdistribution hazard ratio [adj. SHR] 1.62 (97.5% confidence interval [CI] 0.7–3.4), p = 0.30), hypothermia after decannulation was a strong predictor of death (adj. SHR 5.17, 97.5% CI 1.8–14.3, *p* = 0.002) (Fig. 1A–D). In a multivariate competing risk regression model, hypothermia following ECMO decannulation was the strongest factor associated with ICU mortality after ECMO decannulation; while increased lactate levels and need for renal replacement therapy (RRT) were also predictive of mortality (Fig. 1E). Collinearity between RRT and hypothermia was assessed by means of phi correlation coefficient, which within the normal range of 0.12 (*p* = 0.105), indicating no collinearity between these two variables. Surviving patients with post-ECMO-fever had comparable lengths of ICU stay compared to patients without post-ECMO-fever (fever: 10 (5–23) days vs. no fever: 8 (4–16) days, *p* = 0.778). There was a numerical trend towards longer ICU length of stay in surviving patients with hypothermia compared to patients with no hypothermia following ECMO decannulation (hypothermia: 22 (10–35) days vs. no hypothermia: 8 (5–20) days, *p* = 0.078).

**Fig. 1:**
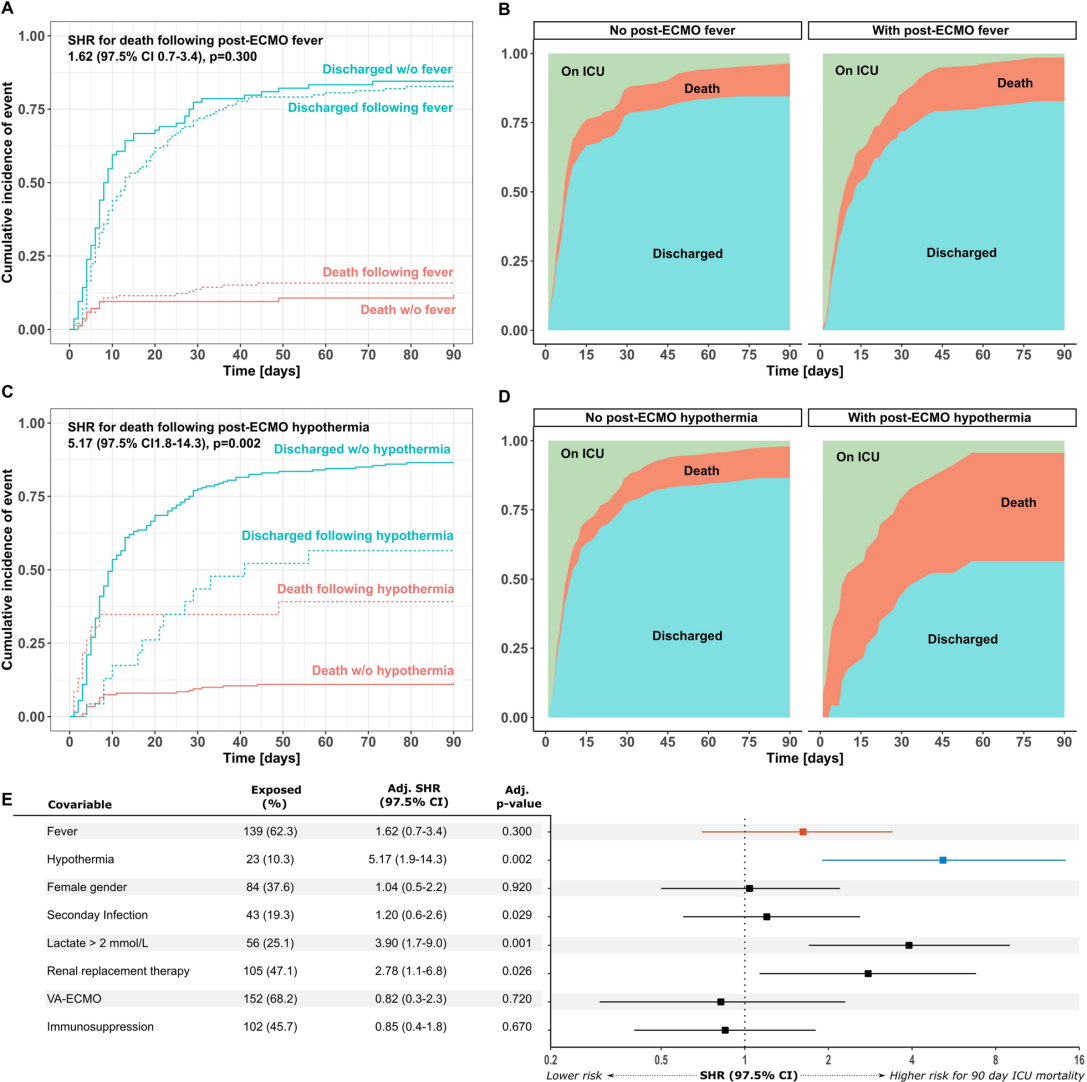
Intensive Care Unit Survival in patients with and without occurrence of fever or hypothermia after ECMO decannulation. Cumulative incidence function of ICU discharge and death (competing event) in patients with and without fever (**A**) and with- and without hypothermia (**C**) following ECMO decannulation. Cumulative incidence of ICU discharge and death as multistate comparison showing no increased incidence of death in patients with fever (**B**), but increased incidence of death in patients with hypothermia (**D**) after ECMO explantation, respectively. The adjusted multivariable competing risk regression model shows hypothermia, increased lactate and need for renal replacement therapy following decannulation as predictive for ICU-mortality **(E)**. Survival is recorded beginning from the time point of ECMO explantation. ICU survival following ECMO decannulation was analyzed for the whole cohort and for the subgroups fever vs. no fever and hypothermia vs. no hypothermia within 72 h following ECMO removal, respectively. Influence of post-ECMO-fever and hypothermia on death on ICU was analyzed by means of a competing risk regression model using death as the primary event and discharge from ICU as a competing event. Post-ECMO-fever or hypothermia was used as a univariable independent risk factor. All reported p-values are two-sided unless indicated otherwise; *p* values < 0.05 were considered statistically significant. We used GraphPad Prism (Version 10.0, GraphPad Software, La Jolla, CA) and IBM SPSS Statistics (Version 25.0, IBM Corp., Armonk, NY) and for data analysis and graph generation. Competing risk regression was performed with the *cmprsk* R package within the R environment for statistical computing version 4.3.1 (R Foundation for Statistical Computing, Vienna, Austria). *SHR* subdistribution hazard ratio, *CI* confidence interval

Several factors, potentially influencing onset of fever, were analyzed in a multivariate logistic regression model. Only increased temperature before ECMO explantation (OR 2.11 (1.08–4.11), *p* = 0.028) was associated with subsequent post-ECMO-fever. Both higher age (OR 0.97 (0.95–0.99), *p* = 0.009) and use of va-ECMO (instead of vv-ECMO) (OR 0.25 (0.11–0.56), *p* < 0.001) were associated with lower incidence of post-ECMO-fever. Neither prophylactic use of single-shot antibiotics nor anti-pyretics before ECMO decannulation were associated with reduced onset of fever. Only need for RRT after ECMO decannulation was associated with increased risk for hypothermia following ECMO explantation (OR 3.25 (1.01–10.48), *p* = 0.049). Higher temperature before ECMO explant (OR 0.15 (0.05–0.45), *p* < 0.001) and again use of va-ECMO (OR 0.24 (0.07–0.8), *p* = 0.021) were associated with reduced risk for later hypothermia.

30/139 (21.6%) febrile patients developed culture positive secondary infections following ECMO explantation (18 bloodstream-infections, eight pneumonias, three wound-infections and one necrotizing-pancreatitis). Neither occurrence of fever nor of hypothermia within 72 h after ECMO were associated with an increased likelihood of culture positive infections. However, fever occurring after 24 h following ECMO decannulation was associated with a higher incidence of infections (OR 2.41 (1.03–5.63), *p* = 0.042). In most cases post-ECMO-fever appears to reflect a sterile inflammation. ECMO implementation itself causes a systemic inflammatory response syndrome (SIRS). Since temperature is throttled under ECMO, it is possible that fever as sign of an ECMO related SIRS only becomes visible shortly after explantation or that a change in hypothalamic set point through external cooling causes fever.

Given the current evidence, moderate fever does not exert a significant negative effect on prognosis in patients in the ICU [4], while in contrast critically ill patients with infections and hypothermia are at high risk for mortality [5].

This study is limited by its retrospective character and the single center setting. The results need further evaluation in larger and prospective multicenter studies.

In summary, we identified post-ECMO-fever as a common but in most cases unharmful condition following ECMO removal while hypothermia may be associated with increased risk of mortality.

## Data Availability

The datasets used and analyzed are during the current study are available from the corresponding author on reasonable request.

## Abbreviations

CI: Confidence interval
ECMO: Extracorporeal membrane oxygenation
HR: Hazard ratio
Hrs: Hours
OR: Odds ratio
RRT: Renal replacement therapy
SIRS: Systemic Inflammatory Response Syndrome
SOFA: Sequential Organ Failure Assessment Score
Va: Veno-arterial
Vv: Veno-venous
Vva: Veno-venous-arterial
